# Defining clinical trial quality from the perspective of resource-limited settings: A qualitative study based on interviews with investigators, sponsors, and monitors conducting clinical trials in sub-Saharan Africa

**DOI:** 10.1371/journal.pntd.0010121

**Published:** 2022-01-27

**Authors:** Angela De Pretto-Lazarova, Claudia Fuchs, Peter van Eeuwijk, Christian Burri

**Affiliations:** 1 Department of Medicine, Swiss Tropical and Public Health Institute, Basel, Switzerland; 2 University of Basel, Basel, Switzerland; 3 Department of Epidemiology and Public Health, Swiss Tropical and Public Health Institute, Basel, Switzerland; 4 Institute of Social Anthropology, Basel, Switzerland; Kerman University of Medical Sciences, ISLAMIC REPUBLIC OF IRAN

## Abstract

**Background:**

Increasing clinical trial cost and complexity, as well as a high waste of clinical trial investment over the past decades, have changed the way clinical trial quality is managed. Recent evidence has highlighted that the lack of a clear clinical trial quality definition may have contributed to previous inefficiencies. This study aims to support the understanding of what clinical trial quality entails from the perspective of resource-limited settings.

**Methodology/Principal findings:**

We conducted 46 semi-structured interviews involving investigators, sponsors, and monitors with experience in conducting clinical trials in 27 countries in sub-Saharan Africa. The questionnaire addressed the overall meaning of clinical trial quality and a conclusive clinical trial quality definition, as well as specific aspects of resource-limited settings across the clinical trial process. We held the interviews either in person, via Skype or by phone. They were recorded and transcribed verbatim, and we performed the analysis using The Framework Method. The analysis of clinical trial quality definitions resulted in 11 elements, which were summarised into a clinical trial quality concept consisting of two components: 1) clinical trial quality building factors (Scientific factors and Moral factors) and 2) promoting factors (Context adaptation; Infrastructure; Partnership; Operational excellence; Quality system). 12 resource-limited settings specific themes were identified. These themes were all categorised under the promoting factors "Context adaptation", "Infrastructure", and "Partnership".

**Conclusions/Significance:**

We found that in order to enable comprehensive clinical trial quality management, clinical trial quality should be defined by a multidimensional concept that includes not only scientific and ethical, but also quality-promoting factors. Such a concept is of general relevance and not limited to clinical trials in resource-limited settings, where it naturally carries particular weight. In addition, from the perspective of sub-Saharan Africa, we identified specific categories that appear to be critical for the conduct of clinical trials in resource-limited settings, and we propose respective changes to a particular existing clinical trial quality framework (i.e., INQUIRE).

## Author summary

In recent decades, the quality management of clinical trials has been criticised for being inefficient and ineffective. This has led to a waste of clinical trial investment and has made it particularly difficult to conduct clinical trials in settings with limited resources. The lack of a universally accepted comprehensive definition of clinical trial quality was suggested as one of the possible causes of inadequate quality management. However, resource-limited countries were not considered in the attempt to create such a definition. In our study, we developed a quality concept based on qualitative interviews from the perspective of investigators, sponsors, and monitors with experience in conducting clinical trials in sub-Saharan Africa. The analysis of these stakeholders’ definitions of clinical trial quality has produced a Clinical Trial Quality Concept that includes quality promoting factors (i.e., Context adaptation; Infrastructure; Partnership; Operational excellence; Quality system) in addition to conventional scientific and ethical factors. The results thus support the need for a multidimensional quality concept to reflect clinical trial quality more comprehensively. We recommend the term “Comprehensive Quality Management (CQM)” for this concept. CQM has the potential to serve as a basis for the current revision of quality management principles in international clinical trial guidelines. Furthermore, the sub-Saharan African perspective has highlighted additional considerations compared to the existing comprehensive INQUIRE clinical trial quality framework. Therefore, we propose including the following three points relevant to resource-limited settings in the framework: 1) Communicating potential infrastructural disadvantages to funders, sponsors, and auditors. 2) Preventing potential exploitation of research populations and workforce in low- and middle-income countries by following existing ethical frameworks. 3) Including "Context adaptation" as an additional framework category (i.e., promoting factor).

## Introduction

The conduct of clinical trials (CTs) in low- and middle-income countries (LMICs) is essential, as it contributes to combatting the burden of poverty-related diseases. Irrespective of the research setting, CTs must meet international standards to assure the public of the participants’ safety and data integrity [1].

Today’s most widely recognised CT guideline is the Good Clinical Practice guideline by the International Council for Harmonisation of Technical Requirements for Pharmaceuticals for Human Use (ICH-GCP) [2]. The guideline is defined as "ethical and scientific quality standard for designing, conducting, recording and reporting trials that involve the participation of human subjects" [3]. However, it is unclear to what extent it is actually linked to the quality of CTs, as its development was criticised for not being evidence-based [4,5]. Moreover, it was developed mainly based on a pharmaceutical industry and regulatory authority perspective from the Global North, which leads to challenges when the guidance is applied to other settings, e.g., non-commercial, academic CTs, or CTs conducted in LMICs [4,6,7].

Also, an overly restrictive interpretation of the ICH-GCP guideline has been associated with decreasing efficiency in CT conduct in the past. Particularly, there was increasing evidence that the practice of assuring CT quality by implementing extensive monitoring was ineffective [1,4,8]. This "reactive" quality management approach had contributed to CT protocols becoming increasingly complex, in turn fuelling the demand for restrictive quality control measures and driving CT costs [9,10]. Consequently, the concept of quality management in CTs was reconsidered in CT guidance, promoting a risk-based approach, which was integrated into a first amendment of the ICH-GCP guideline in 2016 [3]. The ICH guideline is currently being completely revised, with one of the goals being to promote a proactive consideration of quality when designing CT protocols and processes by identifying "Critical to Quality Factors" [11–13].

It has further been demonstrated that as much as 85% of investment in global CT implementation has been wasted [14]. The main reasons were deficiencies in research question selection; study design, conduct, and analysis; CT regulation and management; publication and accessibility of results; and completeness and usability of reporting [15]. These findings highlight that the conduct of CTs has essential quality attributes that go beyond compliance with the current ICH-GCP guideline.

A recent systematic literature survey found that there is no generally accepted consensus on a definition of CT quality [16]. A possible difference in understanding by the various stakeholders involved in CTs about what constitutes quality in CTs can inherently lead to both, undervalued and wasteful activities. Consequently, the authors of that study developed a quality framework called INcreasing QUality In patient-oriented academic clinical REsearch (INQUIRE) to summarise the quality criteria for CTs and achieve broad consensus [17]. The framework also provides an overview of existing standard tools to assure the quality of different parts of a CT (e.g., systematic reviews to define research questions, the SPIRIT statement for protocol development, the CONSORT statement for study publications, following GCP during the CT conduct, monitoring, and auditing). However, for the development of the INQUIRE framework, a LMICs’ perspective was not considered, nor was an openly formulated CT quality definition sought.

Our study aims to provide a complementary viewpoint on the definition of CT quality from the perspective of CTs conducted in sub-Saharan Africa. On the one hand, this perspective allows the identification of challenges of CT conduct in LMICs. On the other hand, the analysis of CT quality in the context of resource-constraint situations may give insight into critical factors contributing to quality and efficiency in the conduct of CTs anywhere in the world.

## Methods

This explorative research follows both an inductive (based on raw data) and deductive (based on concept) theoretical approach. Such an interpretive model represents and involves the interplay of evidence and ideas. By this, collected data are reviewed in terms of theories and concepts and critically reflect on meaningful and reasonable causal relationships, contrasts and similarities, and homogeneity and heterogeneity in view of received meanings, perceptions and notions. The identified significant patterns of meaning are of scientific, practice, or policy relevance. Consequently, the analytical procedure of this study is grounded on The Framework Method according to Gale et al. and its seven steps of data analysis [18].

### Ethics statement

The study was reviewed by the ethics committee Ethikkommission Nordwest- und Zentralschweiz (EKNZ). It was granted an exemption from ethical approval, as it did not fall under the Human Research Act’s remit. The ethics committee confirmed that the project "fulfils general ethical and scientific standards for research with humans and poses no health hazards".

The stakeholders were contacted by email and received an information sheet addressing the study aim, privacy information, and the interviewers’ contact details and credentials. The privacy information included that the interview was voluntary, that withdrawal from the study was possible at any time, and that all identifying information are kept confidential. If they agreed to participate, an appointment was made for the interview. At the beginning of each interview, participants gave verbal consent to participate and their statements to be audio-recorded. We followed the consolidated criteria for reporting qualitative research (COREQ, S1 Table) [19] and verified the trustworthiness of our methods based on criteria described by Anney [20].

### Study design and setting

The study followed a qualitative research approach based on interviews with stakeholders having CT experience in at least one country in sub-Saharan Africa (SSA). The stakeholders could originate from in or outside SSA. Due to the large number of targeted countries, no travelling was intended. The interviewers were based in Switzerland and most interviews were conducted remotely, either by phone or via Skype. Some interviews were conducted face-to-face in Switzerland, with visiting stakeholders.

We focused on three stakeholder groups composed of sponsor representatives (referred to as ’sponsors’), investigators, and monitors with experience in conducting CTs in SSA. These stakeholder groups were selected as they are involved in CT planning, conduct, and quality control and were expected to provide comprehensive knowledge about the entire CT scope.

A semi-structured interview guide using open-ended questions was chosen. It offered the possibility to define the interview’s core components but still allowed the interview participants to address further topics intuitively.

The interviews were conducted by the lead and the second author. Both are pharmacists, female, and acquired interview experience in training and pilot rounds prior to the study. The application of the methods and interpretation of the findings was supervised and reviewed by an experienced social scientist.

### Sampling and recruitment

First, purposive sampling was applied: a list of interventional studies conducted in SSA (excluding South Africa) between 2013 and 2017 was extracted from the WHO International Clinical Trials Registry Platform (ICTRP) [21]. The ICTRP was chosen as it accesses CT data from multiple providers, including ClinicalTrials.gov [22] and the Pan African Clinical Trial Registry (PACTR) [23]. The study period was chosen in order to access stakeholders conducting CTs according to latest ethical guidelines (i.e., the latest update of the Declaration of Helsinki in 2013) and having most current contact details. Also, in earlier study periods, less countries in SSA were involved in the conduct of CTs. South Africa was excluded from the sample to achieve greater diversity of SSA countries, as South Africa accounts for a disproportionately high number of CTs compared to other countries in SSA.

All investigators and sponsor and monitor organisations available in the ICTRP extract were systematically contacted.

Second, as sponsors and monitors in particular were difficult to access through information provided in CT registries, we additionally applied a strategy of snowball sampling: a) We drew on an independent network that we established at international conferences prior to the study. Thereby, we distributed flyers and approached CT stakeholders asking them about their interest in participating in the interview and requesting their contact details. In addition, we screened for contacts based on the conference participant lists. b) We reached out to the Swiss Tropical and Public Health Institute’s (Swiss TPH) professional network.

Interested members of these networks were asked to participate in the interview, as well as to provide us with further contacts until we reached sufficient participants in each stakeholder group. The sample size was defined according to the principle of saturation: Once the interviewers had the impression that no or only a few new contentwise elements were mentioned per stakeholder group, the sample size was considered sufficient. Thereby, the focus was set on defining key elements of CT quality.

While investigators were reached through both sampling strategies (purposive and snowball), sponsors and monitors were mainly reached through snowball sampling (Box 1).

**Box 1. Interview participant recruitment and response rates**

77 potential investigators were contacted based on the ICTRP and previous personal contact:

→ 18 persons accepted, 11 refused, the remaining didn’t reply to the interview invitation

103 potential sponsors and sponsor’s organisations were contacted based on the ICTRP:

→ 1 person accepted, 4 refused, the remaining didn’t reply to the interview invitation

43 potential (known and unknown) monitors and CRO’s were contacted based on the ICTRP:

→ 3 persons accepted, 8 refused, the remaining didn’t reply to the interview invitation

71 potential participants (sponsors, investigators, and monitors) were personal contacts from international conferences and recommendations:

→ 25 persons accepted, 21 refused, the remaining didn’t reply to the interview invitation

47 interviews were conducted in total

→ 1 interview was excluded as the participant only had experience in observational research and not in clinical trials

46 interviews were finally included

ICTRP: International Clinical Trials Registry Platform; CRO: Contract Research Organisation

### Data collection

The semi-structured interview guide (S1 Text) was based on an extensive preliminary literature review to cover the entire CT scope. Nine pilot testing interviews involving staff from the home institution with experience in CTs were conducted to gain information, limit the topic, identify a clear focus of the research scope, and improve the interview guide’s clarity. The participant information sheet and interview guide were written in English and translated into French to extend the range of possible participants. The translation was checked for correct terminology by French- and English-speaking persons.

The following key topics were covered in the interview guide with reference to CTs conducted in SSA: personal and professional background; interpretation of CT quality in general; experience in CT planning, design, initiation, conduct, and termination; collaboration of CT stakeholders; reflection on CT quality; and conclusion based on a one-sentence CT definition.

The interviews were conducted between March and August 2018 and lasted 35–75 minutes. They were recorded using a digital voice recorder (Olympus Digital Voice Recorder VN-733PC), transcribed verbatim, and coded using MAXQDA 2018 VERBI software. During the interviews, the interviewer took notes to reflect and apply minor clarifications to the interview guide for the subsequent interviews. Each audio file was re-listened by a second person, and the interview transcript was double-checked. The data output was anonymised to prevent the identification of interview participants or organisations. Only the lead author has access to the key for data anonymisation.

### Data analysis

The interviews were coded and analysed according to The Framework Method [18].

First, we developed framework matrices for the one-sentence and general CT quality definitions provided by the respondents. The one-sentence CT quality definition matrix served as the basis for defining main categories that form the components of a CT quality concept. The general CT quality definition matrix was used to confirm and complement these categories.

Second, the complete interview transcripts were screened collecting additionally resource-limited settings (RLS) specific themes. These themes were assigned to the CT quality concept categories. The RLS-specific themes were identified by reflecting on which aspects mentioned by respondents across all interview topics dare likely to be influenced by the research setting.

Two authors coded and analysed the one-sentence CT quality definition. The remaining text was coded and analysed by one author and reviewed by a second author. All interviews were re-coded once by the same or a second author. During the coding process, memos on the content were created to support reflections. The codes as well as initial and final categories were reviewed and discussed with the co-authors.

## Results

### Participants

We conducted 46 interviews, including 21 investigators, 13 sponsors, and 12 monitors. Saturation of information was reached after 10–12 interviews. For the investigators’ group, saturation was reached after almost double that number, as a large proportion (n = 8/21) of investigators only had experience in investigator-initiated trials (IITs). Interview participants had experience in 27 countries in SSA and the workplace of more than half of all participants (n = 25/46) was in SSA.

The participant characteristics show some notable differences between groups (e.g., workplace, place of CT conduct, years of experience, research environment). However, the characteristics of the total number of interview participants were relatively balanced (Table 1).

**Table 1:** Interview participant characteristics.

Stakeholder		Gender		Workplace		Place of CT conduct		Av. CT exp.	Research environment		
Type	Number	Female	Male	SSA	Non-SSA	Only SSA	SSA, Non-SSA	Years	IIT	Ind.	Mix
**Investigators**	21	10	11	10	11	11*	7*	9.9	20	9	7
**Sponsors**	13	5	8	4	9	2	11	13.0	3	10	13
**Monitors**	12	5	7	11	1	9	3	13.5	7	9	6
**Total**	**46**	**20**	**26**	**25**	**21**	**22**	**21**	**11.8**	**30**	**28**	**26**

* No response from 3 interview participants; SSA: Sub-Saharan Africa; CT: Clinical trial; IIT: Investigator-initiated trial; Ind.: Industry-sponsored; Mix: Product-development partnership (PDP), Public-private partnership (PPP), or other partnerships; Av. CT exp.: Average CT experience.

The average CT experience of the interview participants was 11.8 years. Most participants were involved in phase II and III CTs and focused on drugs (Table 2). About half of the participants experienced more than one CT phase (n = 24/46) and more than one intervention type (n = 23/46).

**Table 2:** Interview participant experience distribution.

Characteristic		Investigators (n = 21)	Sponsors (n = 13)	Monitors (n = 12)
**Clinical trial experience**	<4 years	4	3	1
	4–9 years	7	2	1
	10–19 years	7	4	9
	20–30 years	2	4	1
	ND	1	0	0
**Clinical trial phase**	I	8	9	7
	II	14	10	12
	III	15	11	11
	IV	6	8	9
**Intervention**	Drug	16	12	12
	Vaccine	11	5	10
	Diagnostic	3	2	3

Many participants (n = 30/46) had CT experience in more than one country in SSA. When considering the registered number of CTs conducted in SSA [20], the countries of conduct were broadly reflected in this study (Table 3). While some countries were slightly underrepresented (Malawi, Nigeria, Rwanda, Cameroon, Zambia), only two (Zimbabwe and Botswana) were clearly underrepresented [20]. As expected, sponsors (5.4) and monitors (4.8) had on average worked in more countries in SSA than investigators (1.7).

**Table 3:** Countries of clinical trial experience.

Countries in SSA (n = 26)*	Number of participants, n
Tanzania	19
Kenya	18
Uganda	13
Burkina Faso	12
Mali	11
Gabon	10
Ghana	10
Mozambique	8
Côte d’Ivoire	7
The Gambia	6
Ethiopia	5
Guinea	5
Malawi	5
Nigeria	5
Senegal	4
Sudan	4
Democratic Republic of Congo	3
Cameroon	2
Rwanda	2
Sierra Leone	2
Zambia	2
Guinea-Bissau	1
4 more countries in SSA**	7

SSA: Sub-Saharan Africa

* Some participants also had experienced CTs conducted in South Africa. However, since South Africa was purposefully excluded in our first step of participant recruitment using the ICTRP, it is underrepresented and, therefore, excluded from this listing.

** Names of countries with low numbers of registered CTs avoided for confidentiality reasons.

### Quality definition

Participants were first asked about their initial thoughts on the meaning of quality in a CT context. Then, the various stages of a CT were run through to create a general overview of the CT process, and in the end, the participants were requested to define the term "clinical trial quality" in *one sentence*. The outcome of this task perfectly summarised the challenge of narrowing this topic down, as many participants (n = 15/46) expressed that this request was challenging or even impossible, and the responses diverged widely and mostly contained multiple aspects:

*"[Laughs] I don’t know, one sentence is really difficult because it’s so much, it’s so many aspects I would say you cannot really define it in one sentence […]."*

    – Investigator, female, 8 years of CT experience, based outside SSA

The original responses can be found in S2 Table. We coded the aspects mentioned by the participants when defining CT quality and grouped them into the following main elements according to descending frequency (Table 4): Data integrity; Adherence; Soundness of research; Participant safety and rights; Quality system; Operational excellence; Partnership; Infrastructure; Relevance and patient-centeredness; Documentation; and Context adaptation.

**Table 4:** Categorisation of the clinical trial quality codes into elements.

Element	Codes
Data integrity	Clinical trial data reflect the clinical trial quality; ensuring that the data are reproducible, verifiable, reliable, solid, credible, authentic, consistent, accurate, good, conclusive, close to reality, trustworthy, excellent, clear, and complete; entering data systematically; safeguarding the data integrity; successful achievement of a correct conclusion from a study
Adherence	Strict/disciplined/rigorous/mandatory adherence to general requirements, such as good clinical practice (GCP), good clinical laboratory practice (GCLP), good manufacturing practice (GMP), and ethical standards, adherence to specific requirements, such as the protocol, standard operating procedures (SOPs), study manuals, and national regulations
Soundness of research	Doing the right; good science; sound scientific premise; no waste; excellent research question; robust study design; clear and simple protocol; looking for the right kind of data; minimization of bias; sound research methodology; sample management; ability to get valid/meaningful/valuable results/correct conclusions/representative figure; meeting the objectives; clear data collection tools
Participant safety & rights	Quality equals safety; protecting/ensuring safety, wellbeing, rights, confidentiality; integrity of volunteers; subjects not put at risk; good participant experience; think about participant safety first; applying all necessary safety measures; having a good safety awareness; no harm
Quality system	A number of aspects should be fulfilled; interaction of multiple factors; totality of data, material and staff; quality is everything/a continuous process/the whole package/a sum of overall implementation; looking at all aspects; a set of factors enabling the collection of data; having robust quality management; steps to ensure quality/consistency; having SOPs at each level; having manuals; ensuring maintenance of instruments; having a risk-management plan; implementing on-site supervision; doing clinical trial administration; keeping control/oversight; having quality control procedures (e.g., implementing monitoring/audits); checking in real-time; picking up errors in time; immediate checks; automatic checks
Operational excellence	Doing it right; doing the best; clever approaches; proper implementation; sophisticated; making an effort; setting the right priorities; smooth/sound clinical trial execution; flexibility; reasonability
Partnership	Desired by site investigator; maintain relationships; networking/exchanging; motivation; communication; meeting the expectations by all parties involved (e.g., sponsors, investigators, local investigators, CRO); alignment of expectations; sharing values, beliefs, and principles; mutual acceptance; taking the community into account; consider the study type; capacity building
Infrastructure	Having a good team; qualified personnel; trained personnel; experience; expertise; dedication; competence; accountability to participants, funders, communities; quality awareness; basic clinical trial understanding (e.g., GCP, GCLP); specific research project understanding; knowing what to do, sticking to the timelines; continuous staff training; mentoring; systems knowledge; variable regulations; adequate facility/equipment/resources
Relevance & patient centeredness^1^	Generating meaningful results for the population (e.g., life improvement of a vulnerable population); having an important research question; utility of the results; putting more focus on the safety than on the publication; having outcomes that people can see; understanding the participants’ needs; addressing the populations’ needs; benefits are rather early than late
Documentation	Trace the research; having a trial master file; having a research protocol; having documented what you have formulated in your case report forms; transparency; having logs
Context adaptation	Adapted to the context (e.g., study environment, population, disease); variable norms (e.g., meaning of blood samples in a cultural context, involvement of the community); consider regional aspects (e.g., weather, politics); adapted to local guidelines and regulations

^1^ This terminology was inspired by the INQUIRE framework as we considered it suitable for the subject [17].

The major consensus we identified, when comparing the CT quality definitions, is that "clinical trial quality" involves multiple layers (n = 10/46).

*"Let’s say, it’s a full quality assurance management system that looks at all the aspects and tries to set up systems for all these aspects, not too heavy, but that can cover many aspects and not only one […]. I mean not just SOP’s [standard operating procedures], it goes beyond SOP’s, it goes…it’s also just […]. Yes, how to cleverly recruit people, how to cleverly follow the data, so, it has several aspects."*

    – Investigator, female, 20 years of CT experience, based outside SSA

While data integrity as well as participant safety and rights, generally ranked among the elements most frequently associated with CT quality, several participants (n = 6/46) referred uniquely to those two broad elements. Half of these participants were sponsors, and half were investigators who had experience in sponsored CTs (commercial or non-commercial).

*"Clinical trial quality is about […] providing assurance that the data you collect is credible and accurate and at the same time making sure that, you know, all the safety, the wellbeing, the rights, integrity of the volunteers are taken into account or are protected."*

    – Investigator, male, over 10 years of CT experience, based in SSA

The elements of data integrity as well as participant safety and rights, were overall most frequently addressed by sponsors, while adherence was most frequently mentioned by monitors, and investigators referred most frequently to the soundness of the research.

When considering additionally the request to describe the meaning of quality in an unrestricted way, the same elements defining CT quality were found. Interestingly, although the overall ranking of elements was slightly variable between the unlimited and the one-sentence CT quality definition, the stakeholder groups mentioning the elements most frequently remained the same, as described above.

**Fig 1:**
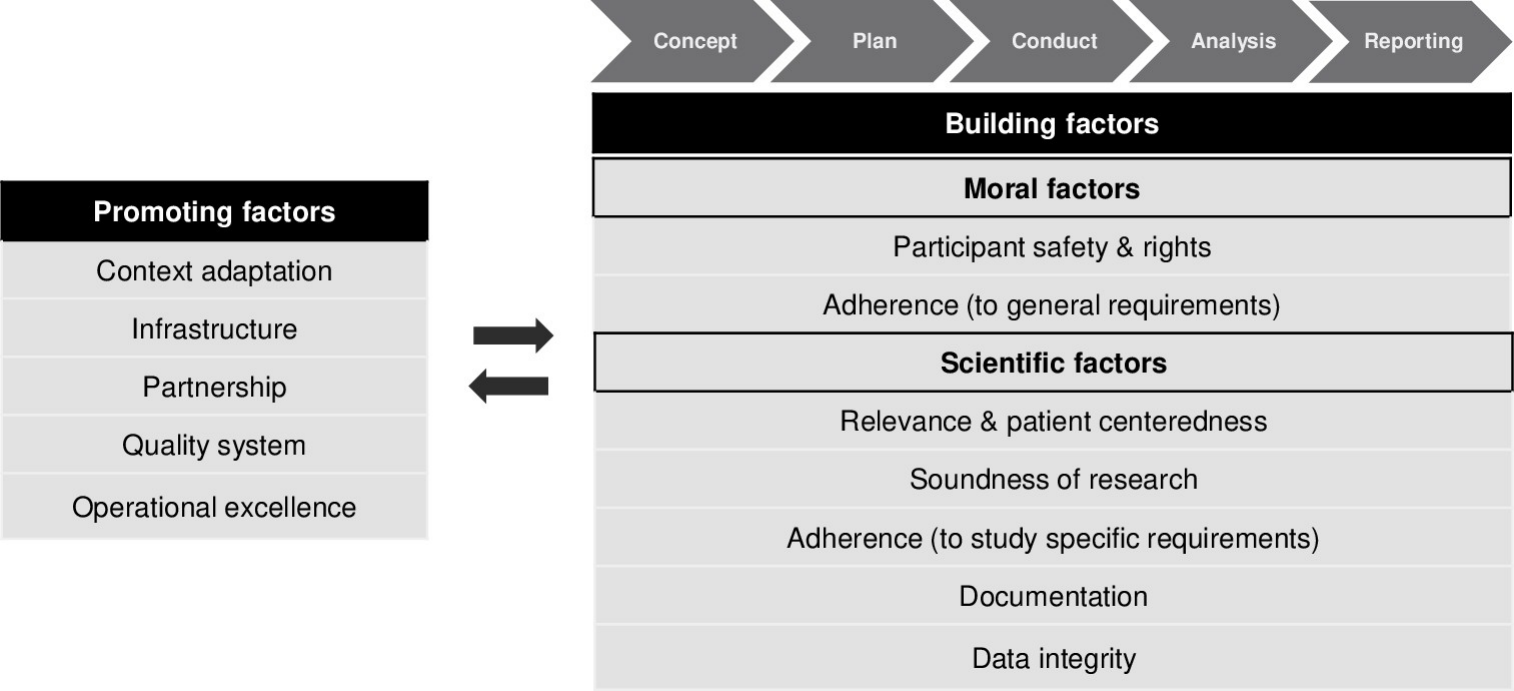
Clinical trial quality concept.

Overall, the 11 CT quality elements could be organised into two components: 1) CT quality building factors covering the entire CT process (i.e., concept, plan, conduct, analysis, and reporting) and 2) CT quality promoting factors. The building factors were further subdivided into i) Moral factors, including the elements "Participant safety and rights"; and "Adherence" to general requirements, and ii) Scientific factors, including the elements "Relevance and patient-centeredness"; "Scientific soundness"; "Adherence" to study specific requirements; "Documentation"; and "Data integrity". The promoting factors include the elements "Context adaptation", "Infrastructure", "Partnership", "Quality system", and "Operational excellence" (Fig 1).

### Resource-limited settings specific themes

When asking about the influence of conducting CTs in resource-limited settings (RLS) on the overall perception of CT quality, some participants first stressed that the quality standard for CTs should be the same, no matter where the CT is conducted.

Nevertheless, participants also indicated that RLS show several characteristics that need to be taken into account to allow the implementation of the same standards. These characteristics could be mainly associated with the CT quality promoting factors "Context adaptation", "Infrastructure", and "Partnership" (S3 Table).

#### Context adaptation

Several interviewees mentioned the need to adapt CT processes to the context relating to the population’s overall health conditions (n = 15/46), accessibility (n = 11/46), and education levels (n = 25/46), as well as to consider cultural (n = 10/46) and regional (n = 7/46) specificities as subthemes.

*Health condition*. Some interviewees highlighted the researchers’ responsibility to consider the generally limited access to healthcare and to manage it ethically (e.g., motivation for CT participation, post-trial treatment access, and appropriate compensation). Also, there is a higher probability of serious co-morbidities and unregistered concomitant medication (e.g., using traditional medicine), which should be addressed, as they could affect patient management and study outcomes. Further, the eligibility criteria should be based on population-specific laboratory norm values, and the development of simultaneous research activities or health intervention programs in the area should be observed.

*"But also it could be, and this is an example […] where […] the local circumstances were such that one of the communities had been heavily researched. It was actually over-researched, and then people are actually tired. So, if you go into that particular area to do a trial again, you may end up with low rates of recruitment […]."*

    – Investigator, male, over 10 years of CT experience, based in SSA

*Accessibility*. Some interviewees mentioned that potential study participants might be difficult to access, particularly in rural areas. They may live far away from health and research facilities, have limited or no mobile phone access, as well as unregistered households. Moreover, neglected diseases may be rare and spread over large areas, requiring adapted recruitment and follow-up approaches (e.g., active recruitment with mobile laboratories).

*Education levels*. Due to limited access to health education, research populations in RLS may have lower awareness about the meaning of research. Some interviewees emphasised that such awareness should be raised before any CT activity is started to prevent rumours and misconceptions (e.g., the meaning of blood, signatures, stigma), as they may seriously impede the recruitment of CT participants. Especially when developing the informed consent process, high illiteracy rates, and the fact that the national language may not be spoken and some local languages may not exist in writing should be considered (e.g., availability of literate impartial witnesses and translators, audio-visual consent tools).

*"Good informed consent is […] the one that can be managed best between being short in text and also complying with all the points that have to appear in an informed consent as of ICH-GCP. […] we more and more go towards audio, audio informed consents. The reality is that in sub-Saharan Africa, in most of the West African countries and East African countries, languages are not written. […] Even if a very literate person speaks well his native language, he can never read it."*

    – Monitor, female, 14 years of CT experience, based in SSA

Overall, many interviewees (n = 15/46) stressed that the informed consent form should be kept short or simple. Some also recommended assessing the participants’ understanding depending on their background knowledge and the research complexity before including them in the study.

*Culture*. Many interviewees (n = 23/46) stressed the importance of engaging community representatives when developing the informed consent process (see also the subtheme Collaboration), as certain aspects may vary according to the cultural context, such as the age of consent, the definition of appropriate representatives to consent on behalf of children, the need of an initial consent by community leaders before addressing individuals, or the appropriateness of group sessions.

*"Usually, for the informed consent, you take the initials of the subject. But in Africa, you can find some people with six to seven names, and the legal representative is also not necessarily the father and mother; an aunt or an uncle could act as a legal representative or elderly people from the same village […]. So, I think an understanding of the culture while you are conducting a clinical trial is also important."*

    – Sponsor, female, over 3 years of CT experience, based outside SSA

*Region*. Some interviewees mentioned that the seasonal disease variability, climatic conditions, political stability, and public holidays might further impede the participant recruitment (e.g., participant reachability) or interfere with internet and mobile phone network connectivity, shipment and transport schedules, as well as storage conditions.

#### Infrastructure

A key topic emerging from many interviews (n = 13/46) when asking about aspects in RLS that affect CT quality were the long timelines that must be considered because of infrastructural shortcomings. Such shortcomings were mainly associated with the subthemes of health authority approval, availability of guidelines, staff qualification, and facility level.

*Health authority approval*. Responses by interviewees clarified that the experience and responsibilities of health authorities may vary widely across SSA. For example, ethics committees may have different requirements (e.g., serious adverse event reporting timelines, inspections, periodic reports, personal or electronic protocol submission). Their approval time may range from two weeks to over one year. Twelve interviewees, who combined had experience in 20 countries in SSA, stressed that national ethics committees’ approval time was often long and unpredictable. Internal bureaucracy and high workload with limited capacity were blamed for the delays. The ethics committees’ diversity was perceived as a particular challenge when conducting multinational trials, and a joint review was recommended. A few interviewees also mentioned that in some countries, the ethics committees’ capacity had improved significantly over the past years and rather considered the regulatory authorities’ capacity one of the most limiting factors. Overall, interviewees recommended anticipating long approval processes in RLS, prioritising, and planning more flexibly.

*Availability of guidelines*. A monitor with experience in about half of all reported countries in SSA (n = 13/27) highlighted that some countries might have established CT guidelines at a national or institutional level, while others may have none. Another interviewee further noted that some countries might be in the process of establishing a research infrastructure; therefore, country-specific requirements may change frequently, and researchers have to adapt to them for every new CT.

All interview participants mentioned that they followed the ICH-GCP guideline. Established guidelines were reportedly mainly based on the ICH-GCP guideline, including additional, country-specific operationalisations.

*"So, some of the recommendations of the ICH-GCP are contextualized in the local guidelines, so, they are really brought to the context of the countries. But they are largely the same, so, we don’t see many divergences from these in critical things."*

    – Sponsor, male, over 3 years of CT experience, based in SSA

Examples of such setting-specific requirements included: reporting frequency to ethics committees; participant compensation; initial community permission; community engagement; blood draws; sample and drug im-/exportation; disease-specific guidance; staff licences (e.g., qualifications for principal investigators/monitors); insurance cover and indemnity; participant type specific guidance (e.g., paediatrics); and informed consent (e.g., age of assent/consent).

*Staff qualification*. When considering staff qualification, interviewees (n = 11/46), who together had CT experience in over two-thirds of the reported countries in SSA (n = 19/27), called out some disadvantages in the education system: The principles of scientific work and GCP were not sufficiently represented during medical training; therefore, in some regions, the graduates were less familiar with, e.g., the concepts of documentation, validation, and deviation. This situation could have consequences for CT quality, as one respondent explained:

*"Medical doctors, investigators, are not trained to use a computer in their routine practice. If you ask them to report […], you face a bottleneck. If you want to go around this and say, ok, we will hire data entry clerks; those data entry clerks can’t read the medical handwritings. They can’t, because these people aren’t trained for research to write them clearly! So, if you want to go around this, too, you will now design very nice source documents, you know, you see where we are going now. We are going to a very tricky, and I call it a poisonous way of doing, but it is now invading clinical research in Africa. You will now design some sort of source document, which is actually a printout of the CRF. Just a data capture tool. The medics and the investigator will just complete that data capture tool and will no more do their medics. They will not do routine practice!"*

    – Monitor, female, 14 years of CT experience, based in SSA

Interviewees also emphasised that inexperienced researchers need time to grow accustomed to these concepts before taking on responsibilities in a CT. One sponsor pointed out the need to start working with an inexperienced site at least one year before implementing the CT. Overall, pilot runs, being initially accompanied by or following experienced researchers from other institutions, and close supervision were considered essential for CT quality when working with less experienced staff.

*Facility level*. Facilities described by interviewees could be very variable and ranged from field labs over primary to tertiary health care facilities to specialized CT units within hospitals or institutional research centres. Urban and rural settings were both common. Most stakeholders had experience using existing facilities, which were frequently upgraded in terms of CT equipment and space. Some (n = 8/46) had also experienced establishing facilities from the ground up.

When working in remote, rural facilities, the coordination of material supply, as well as limited power and internet connectivity, were the main concerns for CT quality due to possible delays. According to interviewees, shipments could involve a lot of bureaucracy in some regions, while the limited internet may require the use of paper Case Report Forms (CRFs), losing the benefits of electronic CRFs, which were summarised by an interviewee as follows:

*"Before, you would have to go through all these papers, and after a few hours, you also get slightly dizzy. But now they have these electronic CRFs that alert you already if there is something out of range […] probably an error that was entered. So, this combination of this electronic CRF, also the way that we now electronically download, e.g., biochemistry and haematology data and upload it into the database prevents a lot of unnecessary query resolution."*

    – Investigator, female, 10 years of CT experience, based outside SSA

Some interviewees (n = 9/46) highlighted the importance of a site assessment visit or questionnaire for an appropriate site selection and study planning. However, a sponsor also warned that formal site assessments were not always desired due to the possibility of harming a centre’s reputation.

#### Partnership

Under the element of "Partnership", CT quality was mainly associated with the subthemes of collaboration, communication, and sustainability.

*Collaboration*. Since various organisations and stakeholders are always involved in the conduct of CTs, interviewees mentioned the importance of good collaboration. Thereby, half of all interviewees (n = 23/46) stressed the importance of community engagement when planning a CT (e.g., involving a community advisory board or community representatives).

*"So, this is the group of volunteers of the community, who sort of act as a communication channel between the clinical trial team and the community. So, those also look at whatever you are planning to do, and they give feedback."*

    – Investigator, male, 7 years of CT experience, based in SSA

Funders may explicitly require community engagement, and in some cases, the ethics committee may be responsible for organising it. Community engagement could also involve research activities to assess the community’s perspective on CTs.

Many interviewees (n = 30/46) emphasised the need to involve site investigators and their team early in the CT development in order to adapt to the site’s working capacity (assessing the need for assistance or extra staff for, e.g., budgeting, accounting, or quality management) and the staff’s routines and schedules (e.g., consider weekends and holidays) as well as to assure protocol and CRF suitability, and particularly to share responsibility.

*"We always involve people in the very early stages of an idea. Because you can have a very nice idea, but if it’s impossible, people are not motivated to do so, it’s not going to work out. So, involving people from sub-Saharan Africa at a very early stage is very important to make sure that they are also owner of the idea and owner of the program. So, ownership is crucial!"*

    – Investigator, male, over 15 years of CT experience, based outside SSA

*Communication*. Good communication was associated with CT quality by many interviewees (n = 36/46). Thereby, interviewees referred to having a communication system between all parties involved in the CT and good communication tools. Some emphasised the notification of important stakeholders about the planning of a CT, reporting to, e.g., ethics committees during the CT, and informing the community about the CT outcomes. For external sponsors and principal investigators, regular and open communication was recommended (e.g., clear definition of roles, communication of expectations, exchange experiences). Moreover, cultural sensitivity was mentioned for the way of communication between the site staff and research participants, as well as between monitors and the site staff. It was also considered crucial for external sponsors to visit research sites to gain a good understanding of the study environment.

*Sustainability*. In some interviews (n = 17/46), it became clear that funding mechanisms, which could also depend on the CT model (i.e., IIT, industry, mixed partnerships), could be very variable and indirectly affect CT quality by influencing capacity building and sustainability. According to interviewees, some funders had clear expectations for CTs (e.g., following ICH-GCP, implementing capacity building, electronic data capturing tools, or community engagement), while others predominantly cared for the CT to deliver results. One interviewee emphasised that applying for competitive grants could particularly result in very limited funding, with only a little flexibility for adjustment. However, flexibility in the budget was necessary for many interviewees (n = 25/46) who had to adjust the funding mainly because, e.g., the CT had taken longer than expected or due to protocol amendments.

Five interviewees raised particular concerns about the importance of adequate core funding and long-term partnership to provide workplace security and sustain research expertise in-between studies.

*"The core funding, and the core support for clinical trial teams is often very limited, and that means also that after one trial that even if it’s only half a year or a one-year break in-between, you might have to start again from scratch because the team could not be paid throughout and they, of course, go somewhere else […]."*

    – Investigator, male, 8 years of CT experience, based outside SSA

Moreover, the establishment of local education programs (e.g., involving students in the CT conduct) and creating more research opportunities by increasing the visibility of research sites, investing in blood-/biobanks, or expanding laboratory capacity were proposed to increase the capacity and sustainability of CT conduct. However, three interviewees stressed that this could only be achieved if governments were more committed. For example, in some countries in SSA, research was considered a "luxury" and therefore as a lower priority.

## Discussion

To the best of our knowledge, this study provides a first overview of the definition of the term "clinical trial quality" by researchers with experience in conducting CTs in RLS. Moreover, it offers an insight into how the research context may contribute to this understanding. The interviewees who represented typical CT models (i.e., IIT, industry, mixed partnerships) in RLS had vast and diverse experience, sometimes in multiple CT models and countries, and could provide comparisons.

Overall, the complexity and multi-layered nature of CT quality, as well as the difficulty of expressing it, led to great variability in its definition (S2 Table). A conceptual interpretation of the definitions was therefore considered the best way to capture the different layers. The resulting quality elements were compiled into a composition of CT quality building (i.e., scientific and moral factors) and -promoting factors (Fig 1). In comparison, these components correspond surprisingly well to the building blocks of the existing INQUIRE framework [17]. On a conceptual level, our results support the defined quality dimensions and the way they span across the different study stages, as presented in the INQUIRE framework. However, variation was found when comparing the CT quality promoters in both quality concepts. In the INQUIRE framework, the CT quality promoters were limited to "Infrastructure" and "Sustainability/Education", while next to "Infrastructure", our concept additionally identified "Context adaptation", "Partnership", "Operational excellence", and "Quality system" as quality promoters. The theme "Sustainability/Education" was thereby classified under "Partnership" in our concept, whereas "Quality system", "Operational excellence", and parts of "Partnership" could be allocated to "Infrastructure" in the INQUIRE framework. Uniquely, "Context adaptation" is a completely new concept that is not reflected in the INQUIRE framework. However, this gap can be explained since the authors of the INQUIRE framework explicitly stated that they did not consider societal aspects and beliefs [16], which are central in our element of "Context adaptation".

When considering the frequency of CT quality elements mentioned per stakeholder group in the one-sentence CT quality definitions, the proportions of monitors mentioning adherence, and sponsors mentioning data integrity as well as participant safety and rights were highest. Investigators mostly mentioned scientific soundness. However, the proportions per element addressed by investigators were distributed more evenly. Overall, the different stakeholder groups tended to focus on the quality aspects most important for their own role. In the case of sponsors, their particular focus on data integrity as well as participant safety and rights might also well be rooted in a perception of CT quality which is influenced by existing guidelines that use exactly these two broad (!) terms to describe what should be aimed for in CTs [3,12]. Awareness about the divergence between the stakeholder groups may promote effective cooperation.

Focusing on RLS-specific factors with potential influence on CT quality, the resulting themes were considered CT quality promoters and could be categorised under "Context adaptation", "Infrastructure", and "Partnership". These themes reflect aspects frequently discussed in the literature. However, existing literature addressed these aspects mainly from the perspective of ethics [24,25], ICH-GCP applicability [2,26,27], or lessons learned when implementing specific studies in specific regions in SSA [28–30]. In contrast, our study was transnational and strictly oriented towards associations with CT quality, which could but did not necessarily have to overlap with these perspectives. We are aware of one other study focusing on quality indicators for CTs in RLS [31]; however, its quality indicator list was restricted to CT implementation rather than the entire CT scope.

When assessing a potential complementary introduction of RLS-specific themes into the INQUIRE framework, there were significant overlaps, indicating that the INQUIRE framework is a very comprehensive tool, even for RLS. Overlaps mainly concerned "Infrastructure" and "Partnership", since "Context adaptation" was not addressed. Still, two aspects should be highlighted that need to be considered in addition to the framework in order to be more inclusive towards RLS: 1) Under "Infrastructure": Emphasis on clearly communicating potential infrastructural disadvantages and their impact on timelines and, ultimately, the quality of CTs to funders, sponsors, and auditors. 2) Under "Sustainability/Education": Prevention of potential exploitation of research populations and workforce in LMICs by following specific ethical frameworks (e.g., *The benchmarks of ethical research in developing countries* [25], and *Good collaborative practice*: *reforming capacity building governance of international health research partnerships* [32]). As a final amendment, we recommend adding 3) "Context adaptation" as an additional CT quality promoter to the INQUIRE framework.

An aspect worth exploring in more detail for an appropriate representation in CT concepts was the distinction or similarity of community engagement versus the engagement of patient representatives. A recent study has shown that there are overlaps but also clear distinctions between both notions [33]. However, in the interviews, it was not clear to what extent these concepts were distinguished or combined under one of the respective terms. Furthermore, it was suggested that the term "patient representative" could be discriminative or exclusive towards research involving healthy populations (e.g., vaccine research).

### Strengths and limitations

This study has some limitations: We focused on the region of SSA, however, the results may not be transferable to all of SSA or RLS outside SSA. Also, we focused only on investigators, sponsors, and monitors without including other CT stakeholders’ perspectives, such as participants, ethics committees, funders, and regulatory authorities. Therefore, further research, including different stakeholder groups and regions, may offer new insights.

Moreover, with our initial sampling strategy using the ICTRP we intended to reach stakeholders independently from each other to maintain confidentiality. However, based on CT registers, mainly investigators and fewer sponsors and monitors could be identified and contacted directly. The overall participation rate remained low, and the following reasons may have contributed to this: Compared to surveys, interviews take much longer, which may have influenced the interest in participation; interviews also require a smaller number of participants, which may have reduced the pressure to participate. In addition, the contacts in the database could be outdated. Therefore, we decided to recruit more participants additionally via the snowball method, whereby a particular selection bias (i.e., personal relationship among interviewees) could hardly be avoided. However, in order to remain as independent as possible, we did not only approach known contacts, but also generated new contacts at conferences.

Further, we intended to increase generalisability by targeting many countries in SSA. which limited us in conducting interviews in person, possibly carrying over this distance into the interview atmosphere. However, since the interviewees were often participating in international research, they were used to the idea of communicating virtually. Also, the topics addressed were not personal, increasing the acceptability of this method. Talking about quality may have also been perceived as a "testing" situation by some participants. We tried to prevent this sentiment by addressing it upfront in the information sheet.

Due to the limited time and budget, we were not able to seek feedback from the interview participants on the interview transcripts and the results. Also, given that many interviews were conducted remotely, we were not able to verify if other persons may have been present in the background during interviews, which may have influenced the responses.

The methodology was qualitative; hence, numerical descriptions should be considered with caution. We also acknowledge that the interviewees’ perspectives may be personal and not generalisable. However, our aim was to look for overarching CT quality aspects and we managed to involve stakeholders with extensive experience, often in several CTs and countries in SSA. Therefore, we believe that the methodology was suitable to produce a usable initial CT quality concept incorporating the perspective from RLS. Nevertheless, the concept could benefit from further research aimed at broader agreement, including transferability to other RLS, transferability to high-income countries, and the inclusion of participant and external feedback.

Finally, our specific methodological approach maintains data trustworthiness by securing the following quality label: this research measures what it should measure (validity); it reproduces reliable results if it will be repeated (reliability); it is objective in the sense that no unwanted influences are exerted by involved persons (objectivity).

### Conclusion

A clear overview of quality components emerged from interviews with investigators, sponsors, and monitors with CT experience in SSA. These components form a CT Quality Concept that, in contrast to the conventional two-dimensional quality standard of the ICH-GCP (R1 & R2), which focuses primarily on scientific and ethical requirements, additionally emphasises "CT quality promoting factors" as a CT quality component.

Our results suggest that considering CT quality promoting factors in the definition of CT quality can lead to a more adequate balance of quality management activities and thus a more efficient and successful CT conduct. This finding is in line with the existing INQUIRE framework, which also represents a multidimensional quality concept and includes the component of quality promoters [17].

Consequently, our results underline that future CT guidelines should not only be founded on two-dimensional but on multidimensional quality concepts, including scientific, ethical, and quality-promoting aspects. We recommend this approach to be understood as "Comprehensive Quality Management (CQM)". CQM could be considered in the ongoing revision (R3) of the ICH-GCP E6 guideline and/or the current draft of the ICH-E8 (R1) guideline and discussed as a basis for the identification of "Critical to Quality Factors" [11–13], enabling a true risk-based approach to CT planning, implementation and oversight.

Insights from the SSA perspective suggested that important considerations in RLS are primarily categorized under the CT quality promoting factors. Hence, when using a traditional two-dimensional quality standard as a basis to manage and assess CT quality, these considerations are neglected, potentially affecting the CT quality and efficiency.

Furthermore, RLS-specific aspects resulted in an additional promoter, Context adaptation, as well as additions to the existing promoters in the INQUIRE framework. Therefore, we open the discussion to include the following points in the INQUIRE Framework to be inclusive towards critical reflections in CTs conducted in LMICs: 1) Addressing potential infrastructural disadvantages and their impact on timelines and, ultimately, the quality of CTs to funders, sponsors, and auditors. 2) Preventing potential exploitation of research populations and workforce in LMICs by following specific ethical frameworks (e.g., *The benchmarks of ethical research in developing countries*, *Good collaborative practice*: *reforming capacity building governance of international health research partnerships*). 3) Adding "Context adaptation" as an additional CT quality promoter category, including reflections on the populations’ socio-economic, as well as cultural and regional aspects.

However, these suggestions are based on exploratory qualitative research. Therefore, they should not be considered exhaustive and need to be validated in further studies.

## Supporting information

S1 Text Interview guides. (DOCX)

S1 Table COREQ. (DOCX)

S2 Table One-sentence clinical trial quality definitions. (DOCX)

S3 Table Coding tree for resource-limited settings specific themes. (DOCX)
