# Supplementary material for: Defining clinical trial quality from the perspective of resource-limited settings: A qualitative study based on interviews with investigators, sponsors, and monitors conducting clinical trials in sub-Saharan Africa
Source: PLoS Negl Trop Dis. 2022 Jan 27;16(1):e0010121. doi: 10.1371/journal.pntd.0010121 (PMC8794119; doi:10.1371/journal.pntd.0010121)
Supplement: S1 Text — (DOCX) [file pntd.0010121.s001.docx]

**S1 Text. Interview guides.**

Interview guide in English

| 1. **Personal and background information** |
| --- |
| 1. Which **country** are you from? 2. Can you tell me about your **professional background**?  - What did you study? - Where did you study? - What is your degree in? - In which institution do you work now? Where?  1. What was your **position** in the conduct of clinical trials?  - How long have you worked in this position? - Have you worked in different positions related to clinical trials? - How long is your overall clinical trial experience?  1. What type/s of **medical intervention/s** have you tested in clinical trials?   (Prompts: drugs, vaccines, diagnostics, etc.)   1. What was/were the **study population/s** in your clinical trials?   (Prompts: children, adolescents, adults, elderly, pregnant, volunteers, patients, etc.)   1. Which clinical trial **phase/s** have you worked on?   (Prompts: I, II, III, IV)   1. How **large** was/were your clinical trial/s in SSA?   (Prompts: population size, mono- vs. multi-centre, duration)   1. In which **country/countries** have you conducted clinical trials (Prompt: in SSA and in non-SSA)? 2. In what type/s of **facility/facilities** did you conduct the clinical trial/was the clinical trial conducted?   (Prompts: hospital, research centre, urban, rural, other)   1. Can you tell me something about the clinical trial **environment** you have worked in?  - Was it an academic, or industrial, or other environment? |
| 1. **General questions about clinical trial quality** |
| 1. I would like to start now with a **general question**: What does clinical trial quality, as a whole, mean to you? 2. Which **factors** do you think have an influence on clinical trial quality? |
| 1. **Questions about quality in clinical trial planning** |
| 1. Can you tell me about how you usually come up with the **idea** of conducting a clinical trial? / What aspects lead to your involvement in a clinical trial conduct?  - Why do you conduct clinical trials in SSA?  1. *If you have experience in clinical trial planning:*  - What are **typical tasks** for you when planning a clinical trial? - How are these tasks **important** for clinical trial quality? (Prompt: what are essential tasks for clinical trial quality during the planning?) - How are these tasks influenced by the fact that the clinical trial takes place in **SSA**?   *If you have experience in more than one country in SSA:*   - Can you tell me more about the **variation** of these tasks from country to country in SSA? - Are there **common** aspects?  1. *If you have experience in different clinical trial sizes*: What effect does the **size** of the clinical trial have on the clinical trial quality? 2. Can you explain more about what effect the **facility** chosen for a clinical trial can have upon the clinical trial quality?  - How did you **choose** the clinical trial facility/facilities? - How **equipped** was/were the facility/facilities? (Prompts: already established facility, new facilities were constructed for the trials)  1. *Referring to the clinical trial environment:* Who was the **sponsor** of your clinical trials? (Prompt: investigator-initiated, university-sponsor, external sponsor, industrial sponsor, consortium, product development partnership?) 2. How was/were your clinical trial/s **funded**? (Prompts: Did you have an external funder *(=funder other than sponsor)*? Did you also have clinical trials with multiple funders?)  - *If you had an external funder/s:*    - What kind of **expectations** did the funder/s have for clinical trial quality?   - In how far did the expectations by the funder **guide** the clinical trial planning and conduct? - Were there any expectations bound to the fact that the clinical trial is conducted in **SSA**? (Prompts: health programs, capacity building, post-trial access to treatment)  1. What **other partners** were involved in clinical trial planning? (Prompts: contract research organisation [CRO], patient representatives, other)  - What kind of **expectations** did they have for clinical trial quality? - In how far did their expectations **guide** the clinical trial planning and conduct?  1. Were you involved in making a clinical trial **budget plan**?   *If yes:*   - On what aspects were the **largest** costs usually budgeted? - Were any **adjustments** made during the clinical trial conduct? On which item were they mostly applied? |
| 1. **Questions about quality in clinical trial design** |
| 1. Speaking further about clinical trial **design**, what are generally important components of clinical trial design? / What makes a **good** clinical trial design?  - What is a good **informed consent**? - What is a good **case report form**?  1. *If you were involved in clinical trial designing:*  - How did you develop the **protocol**? - What **guidelines** did you consider for the clinical trial design? (Prompt: international ones, local ones, CONSORT, ICH-GCP?) - Was the implementation of this/these guideline/s in any way influenced by the fact that the trial is conducted in **SSA**? (Prompt: were there any challenges, when implementing the guidelines?)  1. Can you tell me more about what influence did the ethical committee/s (**EC**s) or institutional review board/s (**IRB**s) have on the clinical trial design?  - **Where** was ethical approval of the protocol received? (Prompts: One country, more countries?)   *In case you have been in contact with a local EC:*   - Can you tell me more about the **reach** of the EC? (Prompt: Was it an institutional, a national, or was it an international one?) - Do you have an idea of the **workload** of this EC? About how much **time** did your approval take? How did this **affect** the clinical trial **design**? How did this **affect** the clinical trial **planning**?  1. How was it decided whether the design was **feasible**? (Prompts: informed consent [e.g., rapid assessment], protocol procedure dry run, case report form [CRF] dry run?) |
| 1. **Questions about quality in clinical trial initiation** |
| 1. Moving now towards **preparatory** steps before clinical trial initiation, what essential tasks had to be completed before the clinical trial/s was/were initiated? (Prompt: What did you have to assure before patient recruitment could be started?)  - Which steps are important for clinical trial quality?  1. How was it assured that clinical trial **staff** (Prompts: monitor, investigator, lab, nurse, pharmacy) is able to perform correctly?  - What **events** took place in this matter? **How** were they trained? **When** were they trained? (Prompts: Was an investigator meeting performed? Was a clinical trial initiation visit performed?) - What influence does **staff experience** have on the clinical trial quality?  1. Was clinical trial initiation influenced in any way by the fact that the trial is conducted in **SSA**? (Prompts: logistics, approvals, contracts, payment schemes) |
| 1. **Questions about quality in clinical trial conduct** |
| 1. Once patient **recruitment** has started, what were typical tasks from your side?  - How are these tasks important for clinical trial quality? (Prompt: what are essential tasks for clinical trial quality during the conduct?)  1. Can you tell me more about how patient **recruitment relates to clinical trial quality**?  - How is patient recruitment influenced by the fact that the trial is conducted in **SSA**? - How does the **informed consent** procedure influence recruitment?  1. How did you assure that everything is **going as planned** during the conduct of a clinical trial?  - Did you implement any specific **quality control**? (Prompt: monitoring?)   *If monitoring was implemented*:   - What **influence** did monitoring have on clinical trial quality? - **How much monitoring** did you apply? (Prompt: was every single aspect checked, or were priorities defined?)   *If priorities were defined:*   - How did you **prioritize**? / Did you **weight** your monitoring/quality control activities according to anything? (Prompt: Risk of the trial, risk of specific events within a trial, risk of specific items within a clinical trial assessed?)   *If other quality control measure was implemented than monitoring:*   - What **influence** did this quality control method have on clinical trial quality?  1. *If "risk" was mentioned:*  - Have you heard or used the **label** risk-based quality management or monitoring? - What are/could be useful **features** of risk-based monitoring? - What could be **challenges** of risk-based monitoring? - To what extent is risk-based monitoring applicable in clinical trials conducted in **SSA**? |
| 1. **Questions about quality in clinical trial completion** |
| 1. What were your typical tasks towards the **end** of a clinical trial?  - How are these tasks important for clinical trial quality? (Prompt: what are essential tasks for clinical trial quality once the last patient has been followed-up?) |
| 1. **Questions about clinical trial quality reflection** |
| 1. Based on what did you **finally judge** the quality of your clinical trial/s? 2. What aspects could have been **improved** in your clinical trials? **Why**? **How** would you improve this/these aspects? 3. What aspects could have been **skipped or shortened**? Is there anything that could have been done more pragmatically? Can you explain why or why not? 4. *If you have experience in both,* ***academic and industrial*** *trials*: what **differences** do you deem important for clinical trial quality? 5. *If you have experience in both,* ***non-SSA and SSA*** *trials:* what **differences** do you deem important for clinical trial quality? 6. Have you experienced **challenges** during the process of a clinical trial you did not foresee?   *If yes:*   - Would you like to share one or more examples from your experience? - What did you do about it/them? - What could have been done to prevent it/them? |
| 1. **Questions about the stakeholders in clinical trial conduct** |
| 1. Would you **recommend** anything to other stakeholders to improve a clinical trial (Prompts: what are lessons learned from the collaborations with monitors? What are lessons learned from the collaboration with investigators*?* What are lessons learned from the collaboration with sponsors*? In case of a consortium*: What are lessons learned from the collaboration with multiple partners?) 2. How may the modes of **payment** be influencing clinical trial quality?  - Based on what have you been paid? (Prompts: regular payments, performance-based payments, e.g., based on patient recruitment) - If monitors were hired: Based on what were they paid? |
| 1. **Conclusions** |
| 1. And moving to the final conclusions: How would you define clinical trial quality in **one sentence**? 2. Are there **any other relevant points** you would like to mention in relation to clinical trial quality? 3. Do you have any questions? |

Interview guide in French

| 1. **Informations personnelles et générales** |
| --- |
| 1. De quel pays venez-vous? 2. Pouvez-vous me parler de votre expérience professionnelle?  - Qu'avez-vous étudié? - Où avez-vous étudié? - Quel est votre diplôme? - Dans quel type d'institution travaillez-vous?  1. Quelle était votre position dans la conduite des études cliniques?  - Depuis combien de temps travaillez-vous dans ce poste? - Avez-vous travaillé dans différentes positions liées aux études cliniques? - Quelle est la durée de votre expérience totale?  1. Quel/s type/s d'intervention/s médicale/médicaux avez-vous testé dans les études cliniques? (Invites: médicaments, vaccins, diagnostic, etc.) 2. Quelle était la/les population/s étudiée/s dans vos études cliniques? (Invites: enfants, adolescents, adultes, personnes âgées, enceintes, volontaires, patients, etc.) 3. Sur quelle/s phase/s d'études cliniques avez-vous travaillé? (Invites: I, II, III, IV) 4. Quelle était la taille de vos études cliniques en Afrique subsaharienne? (Invites: taille de la population, mono- ou multicentrique, durée) 5. Dans quel pays avez-vous réalisé des études cliniques? 6. Dans quel/s type/s d'établissement/installations avez-vous réalisé l'étude clinique / l'étude clinique a-t-elle été effectuée? (Invites: hôpital, centre de recherche, urbain, rural, autre) 7. Pouvez-vous me parler de l'environnement d'études cliniques dans lequel vous avez travaillé?  - Était-ce un milieu académique, industriel ou autre? |
| 1. **Questions générales sur la qualité des études** |
| 1. Je voudrais commencer par une question générale: Qu'est-ce que c’est pour vous, la qualité des études cliniques dans son ensemble? 2. Maintenant, quels facteurs ont une influence sur la/cette qualité des études cliniques selon vous? |
| 1. **Questions sur la qualité dans la planification des études cliniques** |
| On va parler maintenant du processus d’une étude clinique et on commence avec la planification.   1. Pouvez-vous me dire comment vous envisagez habituellement de mener une étude clinique? / Quels aspects entraînent votre participation à une étude clinique?  - Pourquoi menez-vous des études cliniques en Afrique subsaharienne?  1. *Si vous avez participé à la planification d'études cliniques* :  - Quelles sont les tâches typiques pour vous lors de la planification d'une étude clinique? - En quoi ces tâches sont-elles importantes pour la qualité des études cliniques? (Prompt : quels sont les tâches essentielles pour la qualité des études cliniques ?) - Comment ces tâches sont-elles influencées par le fait que l'étude clinique a lieu en Afrique subsaharienne?   *Si vous avez de l'expérience dans plus d'un pays d'Afrique subsaharienne*:   - Pouvez-vous m'en dire plus sur la variation de ces tâches d'un pays à l'autre en Afrique subsaharienne? - Y a-t-il des aspects communs?  1. *Si vous avez de l'expérience dans différentes tailles d'études cliniques*: Comment la taille de l'étude clinique affecte-t-elle la qualité? 2. Pouvez-vous expliquer plus en détail comment l'installation choisie pour une étude clinique peut avoir un impact sur la qualité des études cliniques?  - Comment avez-vous choisi l'installation ou les installations d'études cliniques? - Dans quelle mesure les installations / installations étaient-elles équipées (se référer à l'installation mentionnée)?  (Invites: installation déjà établie, de nouvelles installations ont été construites pour les études)  1. Comment votre / vos étude (s) clinique (s) a / ont été financé (s)? (Invites: Avez-vous eu un bailleur de fonds externe? Avez-vous également eu des études cliniques avec plusieurs bailleurs de fonds?)  - *Si vous aviez un bailleur de fonds externe (= bailleur de fonds autre que le commanditaire):* - Quel genre d'attentes les bailleurs de fonds ont-ils eu pour la qualité des études cliniques? - Dans quelle mesure les attentes du bailleur de fonds guident-elles la planification des études cliniques - Y avait-il des attentes liées au fait que l'étude clinique soit menée en Afrique subsaharienne? (Invites: programmes de santé, renforcement des capacités, accès au traitement après le procès)  1. Quels autres partenaires ont participé à la planification des études cliniques? (Invites: consortium, partenariat pour le développement de produits, organisation de recherche sous contrat [CRO], représentants des patients, autres)  - Quel genre d'attentes ont-ils eu pour la qualité des études cliniques? - Dans quelle mesure leurs attentes ont-elles guidé la planification des études cliniques?  1. Avez-vous participé à l'élaboration d'un plan budgétaire pour les études cliniques?   *Si oui*:   - Sur quels aspects les coûts les plus importants sont-ils habituellement budgétés? - Des ajustements ont-ils été apportés au cours de l’étude clinique? (Invite: sur quel article ont-ils été principalement appliqués?) |
| 1. **Questions sur la qualité dans la conception des études cliniques** |
| 1. Pour en savoir plus sur la conception des études cliniques, quels sont les éléments généralement importants de la conception des études cliniques? / Qu'est-ce qui fait un bon plan d'étude clinique?   - Qu'est-ce qui fait un bon consentement éclairé ?  - Qu'est-ce qui fait un bon formulaire de rapport de cas (CRF)?   1. Si vous-avez participé à la conception d'études cliniques :  - Comment avez-vous développé le protocole? - Quelles directives avez-vous envisagées pour les études cliniques? (Invites : internationale, locale, CONSORT, ICH-GCP ?) - Comment la mise en place des directives a-t-elle été liée au fait que l'étude clinique soit menée en Afrique subsaharienne?  1. Pouvez-vous m'en dire plus sur l'influence des comités d'éthique ou des commissions d'examen international sur la conception des études cliniques?  - Où l'approbation éthique du protocole a-t-elle été reçue? (Invites: Nord, Sud?)   *Au cas où vous seriez en contact avec un comité d'éthique local*:   - Pouvez-vous m'en dire plus sur la portée du comité éthique? (Invite: Était-ce un comité national ou était-ce un comité international?) - Avez-vous une idée de la charge de travail de ce comité? Combien de temps a duré votre approbation? Comment cela a-t-il affecté la conception de l’étude clinique?  1. Comment a-t-on décidé si la conception était faisable? (Invites: consentement éclairé [par exemple, évaluation rapide], protocole, formulaire de rapport de cas [CRF]) |
| 1. **Questions sur la qualité de l'initiation des études cliniques** |
| 1. Passons maintenant aux étapes préparatoires avant le lancement de l’étude clinique, quelles tâches essentielles devaient être accomplies ? (Invite: qu'aviez-vous à assurer avant que le recrutement des patients puisse commencer?)  - Comment ces étapes sont-elles importantes pour la qualité des études cliniques?  1. Comment a-t-on assuré que le personnel des études cliniques (suggestions: moniteur, investigateur, laboratoire, infirmière, pharmacie) est capable de fonctionner correctement?  - Quels événements ont eu lieu dans cette affaire? Comment ont-ils été formés? Quand ont-ils été formés? (Invites: Une réunion d'investigateurs a-t-elle été effectuée? Une visite d'initiation aux études cliniques a-t-elle été effectuée?) - Comment l'expérience du personnel a-t-elle influencé la qualité des études cliniques ?  1. Comment l'initiation des études cliniques a-t-elle été influencée par le fait que l’étude est menée en Afrique subsaharienne? (Invite : logistique, approbations, contrats, modes de paiement) |
| 1. **Questions sur la qualité de la conduite des études cliniques** |
| 1. Une fois que le recrutement des patients a commencé, quelles sont les tâches typiques de votre part?  - Comment sont-elles importantes pour la qualité des études cliniques ? (Invite : Quelles sont les tâches essentielles pour la qualité des études cliniques pendant la conduite?)  1. Pouvez-vous m'en dire plus sur la relation entre le recrutement de patients et la qualité des études cliniques?  - Comment le recrutement des patients est-il influencé par le fait que l’étude est menée en Afrique subsaharienne? - Comment la procédure de consentement éclairé influence-t-elle le recrutement?  1. Comment avez-vous assuré que tout se passe comme prévu?  - Avez-vous mis en place un contrôle de qualité spécifique?   *Si monitoring a été mise en place:*   - Quel impact a-t-il eu sur la qualité des études cliniques? - Combien de monitoring a été mise en place ? (Invite : chaque aspect contrôlé, priorités ?)   *Si des priorités ont été définies:*   - Comment avez-vous priorisé? / Avez-vous pondéré vos activités de surveillance / contrôle de qualité en fonction de quoi que ce soit? (Invite: Risque de l'essai, risque d'événements spécifiques dans un essai, risque d'éléments spécifiques dans un essai clinique évalué?)   *Si autres mesures ont été mise en place :*   - Quel impact ont-elles eus ?  1. *Si "risque" a été mentionné*: - Avez-vous entendu ou utilisé l’étiquette de "surveillance de la qualité basée sur le risque"? - Quelles sont / pourraient être des caractéristiques utiles de la surveillance basée sur les risques? - Quels pourraient être les défis de la surveillance basée sur le risque? - Dans quelle mesure la surveillance basée sur le risque est-elle applicable dans les essais cliniques menés en Afrique subsaharienne? |
| 1. **Questions sur la qualité de la terminaison des études cliniques** |
| 1. Quelles ont été vos tâches typiques vers la fin d'une étude clinique?  - Comment ont-ils influencé la qualité des études cliniques? (Invite: quelles sont les tâches essentielles pour la qualité des essais cliniques une fois que le dernier patient a été suivi?) |
| 1. **Questions sur la réflexion sur la qualité des études cliniques** |
| 1. Sur la base de quoi avez-vous finalement jugé la qualité de vos études cliniques? 2. Quels aspects pourraient avoir été améliorés dans vos études cliniques? Pourquoi? Comment amélioreriez-vous cet/ces aspect/s? 3. Quels aspects auraient pu être ignorés ou raccourcis? Y a-t-il quelque chose qui aurait pu être fait de manière plus pragmatique? Pouvez-vous expliquer pourquoi ou pourquoi pas? 4. Si vous avez de l'expérience dans des études universitaires et industrielles: quelles différences estimez-vous importantes pour la qualité des études cliniques? 5. Avez-vous eu des difficultés au cours d'une étude clinique que vous n'aviez pas prévu? *Si oui:*  - Souhaitez-vous partager un ou plusieurs exemples de votre expérience? - Qu'avez-vous fait à ce sujet? - Qu'est-ce qui aurait pu être fait pour l'éviter? |
| 1. **Questions sur les parties prenantes dans la conduite des études cliniques** |
| 1. Recommanderiez-vous quelque chose aux autres parties prenantes pour améliorer une étude clinique (Invites: quelles sont les leçons tirées des collaborations avec les moniteurs?) Quelles sont les leçons tirées de la collaboration avec les sponsors? Dans le cas d'un consortium: Quelles sont les leçons tirées de la collaboration? 2. Comment les modes de paiement peuvent-ils influencer la qualité des études cliniques?  - Sur la base de quoi avez-vous été payé? (Invites: paiements réguliers, paiements basés sur la performance, par exemple basés sur le recrutement des patients) - Si les moniteurs ont été embauchés: En fonction de quoi ont-ils été payés? |
| 1. **Conclusions** |
| 1. Et passer aux conclusions finales: Comment définiriez-vous la qualité des études cliniques en une phrase? 2. Y a-t-il d'autres points pertinents que vous aimeriez mentionner en relation avec la qualité des études cliniques? 3. Avez-vous des questions? |
