# Supplementary material for: Defining clinical trial quality from the perspective of resource-limited settings: A qualitative study based on interviews with investigators, sponsors, and monitors conducting clinical trials in sub-Saharan Africa
Source: PLoS Negl Trop Dis. 2022 Jan 27;16(1):e0010121. doi: 10.1371/journal.pntd.0010121 (PMC8794119; doi:10.1371/journal.pntd.0010121)
Supplement: S1 Table — (DOCX) [file pntd.0010121.s002.docx]

**S1 Table. COREQ.**

**COREQ (COnsolidated criteria for REporting Qualitative research) Checklist**

A checklist of items that should be included in reports of qualitative research. You must report the page number in your manuscript where you consider each of the items listed in this checklist. If you have not included this information, either revise your manuscript accordingly before submitting or note N/A.

| **Topic** | **Item No.** | **Guide Questions/Description** | **Reported on**  **Page no.** |
| --- | --- | --- | --- |
| **Domain 1: Research team and reflexivity** | | | |
| *Personal characteristics* | | | |
| Interviewer/facilitator | 1 | Which author/s conducted the interview or focus group? | Study design and setting, last paragraph, p7 |
| Credentials | 2 | What were the researcher's credentials? E.g., PhD, MD | Study design and setting, last paragraph, p7 |
| Occupation | 3 | What was their occupation at the time of the study? | Study design and setting, last paragraph, p7 |
| Gender | 4 | Was the researcher male or female? | Study design and setting, last paragraph, p7 |
| Experience and training | 5 | What experience or training did the researcher have? | Study design and setting, last paragraph, p7 |
| *Relationship with participants* | | | |
| Relationship established | 6 | Was a relationship established prior to study commencement? | Ethical aspects, last paragraph, p10 |
| Participant knowledge of the interviewer | 7 | What did the participants know about the researcher? E.g., personal goals, reasons for doing the research | Ethical aspects, last paragraph, p10 |
| Interviewer characteristics | 8 | What characteristics were reported about the interviewer/facilitator? E.g., bias, assumptions, reasons and interests in the research topic | Ethical aspects, last paragraph, p10 |
| **Domain 2: Study design** | | | |
| *Theoretical framework* | | | |
| Methodological orientation and theory | 9 | How were participants selected? E.g., purposive, convenience, consecutive, snowball | Methods section, first paragraph, p7 |
| *Participant selection* | | | |
| Sampling | 10 | How were participants approached? E.g., face- to-face, telephone, mail, email | Sampling and recruitment, p7-8 |
| Method of approach | 11 | How many participants were in the study? | Ethical aspects, last paragraph, p10 |
| Sample size | 12 | How many people refused to participate or dropped out? | Participants, p11 |
| Non-participation | 13 | What were the reasons for this? | Box 1, p8 |
| *Setting* | | | |
| Setting of data collection | 14 | Where was the data collected? E.g., home, clinic, workplace | Study design and setting, first paragraph, p6 |
| Presence of non-participants | 15 | Was anyone else present besides the participants and researchers? | Strengths and limitations, fifth paragraph, p28 |
| Description of sample | 16 | What are the important characteristics of the sample? E.g., demographic data, date | Study design and setting, second paragraph, p7 |
| *Data collection* | | | |
| Interview guide | 17 | Were questions, prompts, guides provided by the authors? Was it pilot tested? | Data collection, first paragraph, p9 |
| Repeat interviews | 18 | Were repeat interviews carried out? If yes, how many? | Box 1, p8 |
| Audio/visual recording | 19 | Did the research use audio or visual recording to collect the data? | Data collection, last paragraph, p9 |
| Field notes | 20 | Were field notes made during and/or after the interview or focus group? | Data collection, last paragraph, p9 |
| Duration | 21 | What was the duration of the interviews or focus group? | Data collection, last paragraph, p9 |
| Data saturation | 22 | Was data saturation discussed? | Sampling and recruitment, p8 |
| Transcripts returned | 23 | Were transcripts returned to participants for comment and/or correction? | Strengths and limitations, fifth paragraph, p28 |
| **Domain 3: analysis and findings** | | | |
| *Data analysis* | | | |
| Number of data coders | 24 | How many data coders coded the data? | Data analysis, last paragraph, p10 |
| Description of the coding tree | 25 | Did authors provide a description of the coding tree? | Table 4 and S2 Table |
| Derivation of themes | 26 | Were themes identified in advance or derived from the data? | Data analysis, first paragraph, p9 and Methods, p6 |
| Software | 27 | What software, if applicable, was used to manage the data? | Data collection, last paragraph, p9 |
| Participant checking | 28 | Did participants provide feedback on the findings? | Strengths and limitations, fifth paragraph, p28 |
| *Reporting* | | | |
| Quotations presented | 29 | Were participant quotations presented to illustrate the themes / findings? Was each quotation identified? E.g., participant number | S1 Table and Results, p13-24 |
| Data and findings consistent | 30 | Was there consistency between the data presented and the findings? | Discussion, p24-30 |
| Clarity of major themes | 31 | Were major themes clearly presented in the findings? | Results, p13-24, Table 4 and S2 Table |
| Clarity of minor themes | 32 | Is there a description of diverse cases or discussion of minor themes? | Results, p13-24, Discussion last paragraph p27 |

**Once you have completed this checklist, please save a copy and upload it as part of your submission. DO NOT include this checklist as part of the main manuscript document. It must be uploaded as a separate file.**

Reference:

1. Tong A, Sainsbury P, Craig J. Consolidated criteria for reporting qualitative research (COREQ): a 32-item checklist for interviews and focus groups. *International Journal for Quality in Health Care*. 2007. Volume 19, Number 6: pp. 349 – 357
