# Supplementary material for: Defining clinical trial quality from the perspective of resource-limited settings: A qualitative study based on interviews with investigators, sponsors, and monitors conducting clinical trials in sub-Saharan Africa
Source: PLoS Negl Trop Dis. 2022 Jan 27;16(1):e0010121. doi: 10.1371/journal.pntd.0010121 (PMC8794119; doi:10.1371/journal.pntd.0010121)
Supplement: S2 Table — (DOCX) [file pntd.0010121.s003.docx]

**S2 Table. One-sentence clinical trial quality definitions.**

| **Interview participant** | **Definition** |
| --- | --- |
| 01A | Well, for me I would call it the clinical trial is the trial that is directly related to the improvement of the life of the vulnerable population. |
| 02A | Let's say, it's a full quality assurance management system that looks at all the aspects and try to set up systems for all these aspects, not too heavy, but that can cover many aspects and not only one. But that does not become too heavy to paralyze the whole trial, because this can be too much and then it can paralyze the whole thing. So it has to be clever, light, but very targeted to the main risks, as you said when we talked about risks, but covering several aspects and not just one, something like that. I mean not just SOP's, it goes beyond SOP's, it goes…it's also just […] Yes, how to cleverly recruit people, how to cleverly follow the data, so it has several aspects. |
| 03A | A good quality trial is a trial that is desired by the local investigators.  …No, I mean, the different thing is, the quality of a trial is, can be assessed by a lot of different things, and maybe not always all together, or, you know, kind of, for me a good quality trial can be a trial where everybody is happy, meaning the sponsor, because he got what he wanted, the CRO because...the investigators they got...the local investigators they got what they wanted...And finally the target population benefits, you know kind of, I think and benefits rather early than rather late, than if it's rather late. |
| 04A | [Laughs] I don't know, one sentence is really difficult because it's so much, it's so many aspects I would say you cannot really define it in one sentence, it's so many interplaying aspects that come together which need to be fulfilled.  […]  I think, I've mentioned several times before it's really these partnerships. It's the presence, the motivation, it's a good team, it's yeah all this. |
| 05A | I cannot, define or...? Yes I think it's difficult to define but...The quality is safeguarding the integrity of clinical trial data and protecting the safety of participants in a clinical trial. |
| 06A | Ahm...In one sentence I would say, you have not done harm and you have documented what you have formulated in your case report forms and you have met your primary objectives. |
| 07A | Based on the quality of the clinical trial, your clinical trials data reflects this quality…and if you want to have good results, valuable results you have to have good quality. |
| 08A | It's a huge challenge [*assumption: to conduct a good quality trial*]…but I think that…it's getting better now, also with the trial master file. Like, my first clinical trial didn't have a master file. So it's getting...now we are getting to more and more rules and stricter which might seem a bit boring and a bit too much when you are stressed in…  […]  Yeah, when you are on the field you are really stressed and you don't want to pay attention on signature. But I think in the end it is really important, to have something that discipline your work step by step, so only have to do this, this, this. |
| 09A | It's a difficult task. Ok, let's try. I would say, check everything on a daily basis to make sure that...Yeah, check everything on a daily basis.  Everything is what you have done as a work, your files, your patient forms, your informed consent form - check your daily work. Take time in the evening to check your daily work, so you ensure the quality. |
| 10A | In one sentence...Clinical trial quality means ensuring that we strictly obey by the step out and that what is outlined in the methodology.  Okay, I would say that the clinical trial quality is ensuring that we strictly obey by the, by the standards that we set out in the methodology. |
| 11A | That's the most difficult question, you are asking me today [laughs].  So, clinical trial quality...Clinical trial quality is a set...I would say it's all factors that help you collect data that will help you arrive at the right conclusions, all the factors that contribute to what...the successful achievement of a correct conclusion from a study. |
| 12A | Clinical trial quality...Yes it is [challenging]. Clinical trial quality...  […]  Ok. Clinical trial quality...ok I can say clinical trial quality is dependent on the quality of data generated, yeah...and ensuring the safety of the participant is upheld at all times. |
| 13A | How I would define it?  Well, you can't [*define quality in one sentence*].  No, in one sentence no. We just talked about it [*referring to the whole interview. Considering also the initial question about the meaning of clinical trial quality, where the participant replied “everything”, we went for “many aspects” in the analysis].* |
| 14A | Yes, I think good clinical trial quality is a clinical trial which follows all ICH-GCP & GCLP requirements and is having a good and well trained team to perform it. |
| 15A | I guess, I would, maybe the...in one sentence...  Let's see: The rigorous execution of an important scientific question downed upon a cohort...or a clinical trial, which stresses an important hypothesis just founded upon a sound scientific premise. They are the phrases brought together in one sentence. |
| 16A | Oh in one sentence [laughs]...Oh gosh, ok, so...I would say in one sentence...Clinical trial quality is about providing or ensuring…providing assurance that the data you collect is credible and accurate and at the same time making sure that, you know, all the safety, the wellbeing, the rights, integrity of the volunteers are taken into account or are protected. |
| 17A | I think clinical trial quality...refers to steps taken, to ensure the smooth running, and minimization of bias and confounding in a trial. |
| 18A | I think I'm going to say: If…clinical trial quality depends on the design and the methodology applied to the study […] But a good quality study is a well-thought, well-planned, well-conducted study even in resource-limited settings. That's what I would say. |
| 19A | In one sentence, the quality means that the compliance with the requirements and the credibility, so that the data which will come out of the clinical trial will be reliable. |
| 20A | The quality after involvement is the safety...wherever the safety is and getting high quality that means that the clinical trial quality, I think, is where you applied very well all...that it takes to the safety of your participants then the quality is there.  I would sum up that it's safety, quality equals safety.  Quality, quality is if you have to carry out a good clinical trial, quality wise you should have a good safety awareness. |
| 21A | Yeah, Clinical trial quality in one sentence is that...the quality is specifically, how good or close to reality, close to the truth you are with your data, in form of a clinical trials, so this would be ability to get accurate and valid information for that kind of study, and that reaches the study objectives. |
| 01B | Quality is equal to...data integrity plus ethics. |
| 02B | I think clinical trial quality is really critical, it's crucial, it's one of the most important aspects and it's really what ensures your data quality and ensures that you think about the patient safety first. All this quality that has to be put in place...it's difficult in one sentence, sorry [laughs]...I think, yeah…quality is a lot about patient safety and wellbeing. |
| 03B | Ok, the quality...the quality of a clinical trial starts from the protocol. The protocol determines the end. If the protocol is complicated, then the study tools may not be clear enough to collect the kind of data that you need to answer the research question, so if the protocol is also ambiguous, then, you know, your analysis of the statistics may not be clear enough, you know, to define the end points. I think, for me it's really the protocol. The simpler the protocol, the lower the risk of having problems with quality and implementation. So more complex protocols, more ambiguous protocols, you know, risk of you having problems with quality down the stream. |
| 04B | Integrity of data...So no matter what kind of results we get, we have to be able to reproduce by doing different analyses at the end of the day. The quality...most of the people...That's all, that's with the integrity of the data at the end. |
| 05B | Wow...hm. Ok, I would say a trial that has been conducted adhering to the protocol, while ensuring the safety of the trial participants. |
| 06B | Mmh, that's a good question…well, I suppose, the bottom line is. I would...You can say that the quality of the clinical trials was good, if the data are good. I mean that's...it's all about the data, unfortunately, at the end of the day it is the data. |
| 07B | Oh my…in one sentence...clinical trial quality really comes down to good participant experience and trustworthy data. |
| 08B | You are aspiring to demonstrate operational excellence as well as excellent quality of data but you have to be flexible to understand that they will not necessarily be perfect, and you will need to adapt and be accepting of what you find with a reason. |
| 09B | Quality data is data that we can depend on to answer our question. To me that's what it means.  Clinical trial quality, yes, it is thrilled down to data eventually, because this is what we are trying to generate. |
| 10B | I would say...I would define clinical trial quality and that it's not well defined. I think particularly in African setting that whole quality management of clinical trials…that is something that needs to be developed as an independent unit so that it supports the trials better…and I don't see enough networking of quality people in Africa to see that they help each other very well. |
| 11B | Clinical trial quality are all the measures that are put in place in order to protect subjects who take part in clinical trials while ensuring meaningful data generation. |
| 12B | Clinical trial quality in once sentence...well...You need a sound protocol and an adequately trained team and taking into account the community and patients' wellbeing into consideration. |
| 13B | Wow. I think for a good clinical trial it is important that the data that will come out of it is clear and the patient is not put at risk. |
| 01C | I would not be able to define it in one sentence, and I will tell you why [laughs]. So I am currently dealing, or participating in a study where...which I believe was messed up by a monitor and the quality was really bad, so then our CRO was contracted to come in and try to mitigate and the quality extremely low to an extent that even enrolment logs were missing. So quality of clinical trials is that monitors' objectives still vary in sub-Saharan Africa, I would say. Largely, the quality is good, but there is still, you know, projects of very poor quality of the research than under the watch of monitors actually. So there's still quite a bit work that needs to be done. |
| 02C | In one sentence, oh...The quality of the study is the totality of data, material, and staff that collaborates to reach this quality. |
| 03C | In one sentence! I can't say in one sentence; I always touch a lot of whatever I have been mentioning. Maybe I would say in one word, compliance to protocols, procedures, GCP/GCLP and ethical requirements. |
| 04C | Clinical trial quality is mandatory, it's everything...  I would say clinical trial quality is not...is not…something that people could say I want to do it, I don’t want, it’s mandatory. If we are not adhering to the guidelines, then nobody is going to trust what we are producing. So the quality is mandatory, no discussion on quality, I say no discussion on quality, no discussion! |
| 05C | Yes I’m saying that the quality of a clinical trial must be a trial that is conducted according to the laid out principles, as ensured in the ICH-GCP standards and according to SOPs and according to the protocol. |
| 06C | How will I define quality? Quality is…quality is doing what you do best, sticking with the international standards. |
| 07C | Ah…mmm! I think that’s the most difficult one!  […]  Ok. So I will say…yeah…quality is a continuous process [laughing]. |
| 08C | Clinical trials practice is a […] set of the GCP-ICH guideline, country regulation guideline and sponsor, and site SOP. |
| 09C | Yeah...in one sentence [laughing]...You know, the thing is that at the end of the day, the clinical trial should be able to give you the right figure, you know, the right figure of the question you are asking yourself. You need to ensure that when you are doing a clinical trial, the information you will have is the right information. I mean you need to get the right information when you are doing a trial. You do not need to have a bias, you do not need to have a, you know...a good clinical trial is a clinical trial that gives you the right information. |
| 10C | For my expertise, doing the right thing at the right time [mentioning earlier in the interview: doing the right things right]. |
| 11C | I think there are a lot of challenges, but every person involved with this standardization, with ICH-GCP, every person is fighting to fulfill all the requirements of ICH-GCP. I think this is a good thing. |
| 12C | Oh my! [laughs]. Ah…Clinical trial quality…I would say that clinical trial…  I mean for me just clinical trial quality means to stick to the protocol and guidelines that are required. I mean I can't think of anything else of like a summary for that. They have to follow. |

A: Investigator; B: Sponsor; C: Monitor
