# Supplementary material for: Defining clinical trial quality from the perspective of resource-limited settings: A qualitative study based on interviews with investigators, sponsors, and monitors conducting clinical trials in sub-Saharan Africa
Source: PLoS Negl Trop Dis. 2022 Jan 27;16(1):e0010121. doi: 10.1371/journal.pntd.0010121 (PMC8794119; doi:10.1371/journal.pntd.0010121)
Supplement: S3 Table — (DOCX) [file pntd.0010121.s004.docx]

**S3 Table. Coding tree for resource-limited settings specific themes.**

| **CT quality promoting factors** | **Themes** | **Codes** | **Subcodes** |
| --- | --- | --- | --- |
| Context adaptation | Population differences | Health condition | Participant compensation as frequent issue in SSA |
|  |  |  | Represent the population flexibly |
|  |  |  | Motivation for CT participation |
|  |  |  | Conflicting health system developments |
|  |  |  | Concomitant diseases |
|  |  |  | Population type |
|  |  |  | Concomitant medication |
|  |  |  | Over-researched population |
|  |  | Accessibility | Finding patients for follow-up can be tricky |
|  |  |  | Recruitment strategy |
|  |  | Education | Awareness |
|  |  |  | Rapid assessment of understanding |
|  |  |  | Trust |
|  |  |  | Language |
|  |  |  | Not written language |
|  |  |  | Illiteracy |
|  |  |  | Translating |
|  |  |  | Simple ICF |
|  |  |  | Short ICF |
|  |  | Culture | Initial consent by community leaders/community |
|  |  |  | Age of consent |
|  |  |  | Appropriate representatives |
|  |  |  | Meaning of blood |
|  |  |  | Importance of culture |
|  | Regional aspects | Region | Public holidays |
|  |  |  | Seasonality |
|  |  |  | Politics |
|  |  |  | Weather problems |
| Infrastructure | Capacity | Processes take more time | RA approval takes more time |
|  | Health authority approval | ECs example | Different requirements |
|  |  |  | Approval time by EC |
|  |  | EC approval long & unpredictable | Reasons for delays (internal inefficiencies, limited capacity, high workload) |
|  |  | Harmonize laws in West Africa | Joint review |
|  |  | Latest developments | Submission process improved |
|  |  | Recommendations | Anticipation, prioritisation & flexibility recommended |
|  | Availability of guidelines | National laws and guidelines / International regulations | Followed ICH-GCP |
|  |  |  | Disease specific guidelines |
|  |  | Local guidelines | No locally modified guidelines |
|  |  |  | Institutional guidelines / Specific local requirements |
|  |  |  | In process of establishing guidelines |
|  |  |  | Mainly based on ICH-GCP |
|  | Staff qualification | Disadvantages in education system | Adequate training time |
|  |  |  | Measures (pilot, close monitoring, accompany) |
|  | Facility level | Facility characteristics | Variable facility types |
|  |  |  | Urban vs. rural |
|  |  |  | Established facilities themselves |
|  |  | Challenges with remote, rural location | Supply-chain bureaucracy |
|  |  |  | Power and internet connectivity |
|  |  | Site assessment visit / Questionnaire | Reputation |
| Partnership | Collaboration | Local partners | Variable |
|  |  | Community engagement | *Community consent (see Culture)* |
|  |  | Local PIs involved (feasibility) | Early involvement in protocol development |
|  |  |  | Routine & Schedule |
|  |  |  | Share responsibility |
|  | Communication | Good communication | Having a communication system for all parties |
|  |  |  | Informing important stakeholders |
|  |  |  | Open communication channel between sites |
|  |  |  | Consider cultural differences |
|  |  |  | Sponsors to engage with local collaborators |
|  | Sustainability | Funding mechanisms | Variable funding |
|  |  |  | Flexibility in budget |
|  |  |  | Long-term partnership |
|  |  | Capacity building | Education opportunities |
|  |  |  | Research opportunities |
|  |  |  | Empowerment |
